# Body Image disturbance in patients with cancer

**DOI:** 10.1192/j.eurpsy.2022.1009

**Published:** 2022-09-01

**Authors:** A. Guermazi, S. Hentati, F. Guermazi, R. Masmoudi, I. Baati, I. Feki, J. Masmoudi

**Affiliations:** Hedi Chaker University Hospital, Sfax, Tunisia, Department Of Psychiatry A, Sfax, Tunisia

**Keywords:** chemotherapy, cancer, body image, Body Image Scale (BIS).

## Abstract

**Introduction:**

Cancer and its treatments have been shown to have a negative psychological effect on many cancer patients. One of these effects is often described as body image disturbance.

**Objectives:**

To assess body image in cancer patients and its association with clinical variables.

**Methods:**

This was a cross-sectional study, conducted over 1 month, involving 100 cancer patients followed in the oncology department at the Habib Bourguiba University Hospital in Sfax (Tunisia). All participants completed a 10-item Body Image Scale (BIS) questionnaire to assess body image dissatisfaction.

**Results:**

These results showed that half of the patients were female, and 70% of them were married. Their mean age was 51.96 years with extremes ranging from 41 to 60 years. Their level of education did not exceed primary school in 61% of cases, and 68% of them were unemployed. A total of 58% of patients received chemotherapy and 44%, 25%, and 11% of persons were affected by breast, digestive and cavum cancer, respectively. Impaired body image was noted in 81% of cases with an average BIS score of 15.39. An altered body image was statistically correlated with female sex (p = 0.005), absence of professional activity (p = 0.032), and the presence of anxiety-depressive symptoms (p = 0.008).

**Conclusions:**

In this study most of the cancer patients had body image disturbances. Therefore, it is to the health team and nurses that take the concept of body image more serious and make use of some interventions to minimize the possible side effects.

**Disclosure:**

No significant relationships.

